# Genetic Polymorphisms of GGH and ABCC2 Are Associated with Methotrexate Intolerance in Patients with Rheumatoid Arthritis

**DOI:** 10.3390/jcm10184070

**Published:** 2021-09-09

**Authors:** Alejandro Escudero-Contreras, Clementina López-Medina, Eduardo Collantes-Estévez, Rafaela Ortega-Castro, Jerusalem Calvo-Gutiérrez, Natalia Mena-Vázquez, Blanca Panero-Lamothe, Bárbara Manzanares-Martín, Rafael Cáliz-Cáliz, Alberto Jiménez-Morales, Mayte Ruiz-Jiménez, Pilar Font-Ugalde

**Affiliations:** 1 Rheumatology Department, Reina Sofia University Hospital, 14004 Cordoba, Spain; alexcudero2@gmail.com (A.E.-C.); educollantes@yahoo.es (E.C.-E.); orcam84@hotmail.com (R.O.-C.); yeru83@hotmail.com (J.C.-G.); fougp@hotmail.com (P.F.-U.); 2 Maimonides Institute of Biomedical Research of Cordoba (IMIBIC), 14004 Cordoba, Spain; barbara.manzanares.sspa@juntadeandalucia.es; 3 Medical and Surgical Sciences Department, University of Cordoba, 14004 Cordoba, Spain; 4 Institute of Biomedical Research from Malaga (IBIMA), 29010 Malaga, Spain; nataliamenavazquez@gmail.com (N.M.-V.); blanca.panero@gmail.com (B.P.-L.); 5 Rheumatology Department, Regional University Hospital from Malaga, 29010 Malaga, Spain; 6 Immunology and Allergology Department, Reina Sofia University Hospital, 14004 Cordoba, Spain; 7 Rheumatology Department, Virgen de las Nieves University Hospital, 18014 Granada, Spain; rcalizcaliz@gmail.com; 8 Pharmacy Department, Virgen de las Nieves University Hospital, 18014 Granada, Spain; alberto.jimenez.morales.sspa@juntadeandalucia.es; 9 Medical Science Liaison Roche, Roche Farma, 28042 Madrid, Spain; mayte.ruiz_jimenez@roche.com

**Keywords:** methotrexate, rheumatoid arthritis, genetics, biomarkers

## Abstract

Objective: to identify new single-nucleotide polymorphisms (SNPs) in genes encoding proteins involved in methotrexate (MTX) metabolism and to evaluate the associations of these SNPs with MTX toxicity or intolerance in a southern Spanish cohort of patients with rheumatoid arthritis (RA). Methods: An observational, retrospective, and multicenter study was conducted at three participating hospitals in southern Spain. The main variable was intolerance to MTX (i.e., bDMARD monotherapy), defined as an interruption of treatment due to adverse events or toxicity. Patients being treated with MTX and bDMARDs (combined treatment) at the time of the study visit were considered “tolerant” of MTX. Ten polymorphisms were selected for sequencing in our patients according to a literature review. Each polymorphism was classified according to three possible genotypes (e.g., two homozygous (AA or GG) and one heterozygous (AG)), and the association of these combinations with MTX intolerance was evaluated. Results: A total of 227 patients were included in the final analysis (107 intolerant of MTX and 120 tolerant). A significant association was observed between MTX intolerance and the GGH-T401C AA/AG genotype (OR 2.13, 95% CI 1.06–4.29) in comparison with the GG genotype. On the other hand, an inverse association was observed between the ABCC2-C24T TT/TC genotype and intolerance to MTX (OR 0.59, 95% CI 0.35–1.00) in comparison with the CC genotype. Conclusion: This study provides new data on the association between genetic polymorphisms and MTX intolerance, which may contribute to the development of new biomarkers and personalized medicine in patients with RA.

## 1. Introduction

Rheumatoid arthritis (RA) is an autoimmune rheumatic disease characterized by synovial inflammation and cartilage damage, which leads to joint destruction and mobility limitations in patients receiving inadequate treatment [1].

Although RA is a chronic disease, knowledge of its pathogenesis has facilitated the development of new drugs that are increasingly effective. Both the European League Against Rheumatism (EULAR) and the American College of Rheumatology (ACR) recommend the use of nonsteroidal anti-inflammatory drugs (NSAIDs) and conventional synthetic antirheumatic drugs (csDMARDs) as first-line treatment in patients with active disease as soon as possible, and both organizations agree on the use of methotrexate (MTX) as the first drug of choice [2,3]. MTX can be used as monotherapy or in combination with other DMARDs (such as biological DMARDs (bDMARDs)) or glucocorticoids. In fact, according to these recommendations, if the treatment target is not achieved after MTX, the addition of a bDMARD can be considered (combination therapy). However, approximately half of patients do not achieve the treatment target, and approximately 20% of patients interrupt MTX treatment due to the appearance of side effects or intolerance [4,5,6].

Polymorphisms in genes encoding transporters and enzymes mediating the biotransformation and elimination of MTX have been suggested as one cause of adverse events [7]. In fact, several studies have shown that two single-nucleotide polymorphisms (SNPs) (rs1891133 and rs1801131) in the 5,10-methylenetetrahydrofolate reductase (MTHFR) gene, which is involved in the intracellular MTX pathway, are associated with MTX toxicity [8,9,10]. Similarly, a polymorphism (rs1051266) in the SLC19A1/RFC180GA gene, a member of the solute carrier (SLC) family of uptake-type transporters that is involved in MTX cell entry, is associated with MTX treatment toxicity in the European population [9]. In addition, the SNP-C3435T (rs104562) in ABCB1, which encodes a membrane glycoprotein, is associated with MTX intolerance [11].

Despite the extensive study of these gene polymorphisms, no reliable biomarker has been identified to predict MTX intolerance or toxicity in patients with RA. Personalized medicine focuses on the identification of SNPs present in transporters and enzymes mediating the elimination of MTX and might provide valuable information to predict MTX intolerance and toxicity in these patients.

Therefore, we decided to conduct this study with the aim of identifying new SNPs in genes encoding proteins involved in MTX metabolism and to evaluate the associations of these SNPs with MTX toxicity or intolerance in a southern Spanish cohort of patients with RA.

## 2. Materials and Methods

### 2.1. Study Design and Patients

This observational, retrospective and multicentre study was conducted at 3 participating hospitals in southern Spain (University Hospital Virgen de las Nieves from Granada, Carlos Haya University Hospital from Málaga and University Hospital Reina Sofía from Córdoba). Consecutive patients with RA fulfilling the following inclusion criteria were selected for this study: (a) diagnosis of RA according to the ACR/EULAR criteria [12], (b) current bDMARD treatment at the time of the inclusion visit (infliximab, etanercept, adalimumab, golimumab, tocilizumab, abatacept, or rituximab), (c) MTX treatment at any time during the course of the disease, and (d) signature on the informed consent form.

Patients receiving MTX and bDMARD treatment (combined treatment) at the time of the study were considered “tolerant” to MTX. Patients receiving bDMARD monotherapy at the study visit were asked about the reason for MTX discontinuation. In cases of discontinuation due to adverse events or toxicity (such as nausea; vomiting; dyspepsia; alopecia; oral ulcers; leukopenia; hepatic alterations, defined as alanine aminotransferase levels greater than 1.5 times the upper normal limit; or pulmonary toxicity), these patients were considered “intolerant” to MTX. Investigators were asked if the intolerance was caused by the MTX and confirmed the resolution of the adverse event after MTX withdrawal to ensure that the intolerance was due to MTX. In the case of MTX discontinuation for reasons other than toxicity (i.e., improvement, inefficacy, and contraindication), the patient was not included in the analysis.

All patients provided written informed consent, and the study was approved by the ethics committees of the three hospitals.

### 2.2. DNA Extraction and Genotyping

Eight polymorphisms in seven genes encoding proteins involved in MTX metabolism (pharmacokinetics and pharmacodynamics) and MTX toxicity, as well as two polymorphisms located in noncoding sequences, were selected for sequencing in our patients according to a literature review. The analyzed polymorphisms were involved in:

- Active transport of MTX: RFC-1-G80A (SMP rs1051266);
- MTX polyglutamate formation: GGH-T401C (SNP rs11545078);
- Folate cycle and purine synthesis: MTHFR-C677T (SNP rs1801133), MTHFR-A1298C (SNP rs1801131), DHFR (SNP 1105525), SHMT1-c1303C > T (SNP rs1979277) and ITPase-C94A (SNP rs34743033), and the latter is located in the noncoding sequence;
- MTX extraction pumps: ABCC2-C24T (SNP rs717620), ABCB1-c3435C > T (SNP rs1045642) and SLCO1B1 (SNP rs11045879), the last of which is located in the noncoding sequence.

Genomic DNA was obtained from blood samples collected during the previous 5 years before the initiation of the study and stored at each hospital. However, DNA was extracted from saliva samples obtained from patients at the University Hospital Virgen de las Nieves using a buccal swab and the QlAamp DNA Mini kit (Qiagen GmbH, Hilden, Germany) according to the purification protocol provided by the manufacturer and stored at −40 °C.

The 10 polymorphisms were sequenced using a real-time polymerase chain reaction system for the discrimination of alleles using TaqMan^®^ probes (7300 Real-Time PCR System, ABI Applied Biosystems, Bedford, MA, USA). The analysis was performed according to the manufacturer’s instructions. Allelic variants (i.e., adenine (A), cytosine (C), guanine (G), and thymine (T)) were determined using StepOne v2.3 software (Applied Biosystems, Bedford, MA, USA).

### 2.3. Variables

This study involved two different variables: one is intolerance to MTX (i.e., bDMARD monotherapy), defined as an interruption of treatment due to adverse events or toxicity, and the other is tolerance to MTX (i.e., bDMARDs and MTX combined treatment) at the time of the study visit.

In addition to the polymorphisms, the following variables were collected:

- Sociodemographic characteristics: age and sex;
- Disease-related variables: disease duration (months), age at diagnosis, time since bDMARD initiation (months), and duration of MTX treatment (months);
- Disease activity at the time of the study visit: c-reactive protein (CRP) in mg/dL, erythrocyte sedimentation rate (ESR) in mm/h, number of tender joints (NTJ), number of swollen joints (NSJ), physician assessment (visual analogue scale ranging from 0–10 points), patient assessment (visual analogue scale ranging from 0–10 points), Disease Activity Score 28 (DAS28), Simple Disease Activity Index (SDAI), and Clinical Disease Activity Index (CDAI).

Among patients belonging to the “intolerant” group, the reasons for MTX withdrawal and adverse events were recorded.

The study was approved centrally by the Ethics Committee at the Reina Sofia University Hospital (protocol code RCC-MTX-2016-01), and each participant signed an informed consent form before inclusion in the study.

### 2.4. Statistical Analysis

The sample size was estimated considering an alpha risk of 5% and a power of 80% to detect a minimum odds ratio of 2.2 for the presence of polymorphisms associated with toxicity or intolerance to MTX. According to the hypothesis that the polymorphism rate would be 50% in the group of “intolerant” patients (i.e., bDMARD monotherapy) and a 3:1 inclusion ratio, the minimum estimated sample size was 251 controls (tolerant patients) and 93 cases (intolerant patients).

Qualitative variables were compared between the two groups (i.e., tolerant vs. intolerant) using the chi-square or Fisher test, and continuous variables were compared using the *t*-test or Mann–Whitney test.

All genetic variants were tested for deviation from Hardy–Weinberg equilibrium. Each polymorphism was classified according to the three possible genotypes (e.g., two homozygous (AA or GG) and one heterozygous (AG)), and the association of these genotypes with MTX intolerance was evaluated using the chi-square test.

Finally, alleles of each polymorphism were classified into two groups according to the presence of one base and using the homozygosity of the other base as a reference. For example, SNPs with homozygous AA and GG and heterozygous AG genotypes were grouped as AA vs. GG/AG to determine the association between MTX intolerance and the G base. These associations were tested through univariate and multivariate logistic regression analyses using the variable “tolerant/intolerant” as the dependent outcome and the polymorphisms as explicative variables. Goodness of fit was evaluated with the Hosmer–Lemeshow test. All the contrasts were bilateral, and a *p*-value < 0.05 was considered significant.

Statistical analyses were conducted by RAIER (Andalusian Network in Rheumatology Research) using SPSS v25 software(IBM, Chicago, IL, USA) Clinical data were collected using the Research Electronic Data Capture (RedCap) system (www.project-redcap.org, accessed on 6 September 2021), and the reporting of this study conforms to the STROBE statement [13].

## 3. Results

Among the 255 patients with RA selected for the study, 28 were excluded because the reason for MTX withdrawal was not toxicity or intolerance. Thus, 227 patients were included in the final analysis, among whom 107 were intolerant of MTX (i.e., patients receiving bDMARD monotherapy) and 120 were tolerant of MTX (i.e., patients receiving bDMARD and MTX combination therapy). The mean age was 60.0 (12.1) years, and 78.4% were female. The mean age at RA diagnosis was 44.2 (12.2) years, and the mean disease duration was 1.48 (7.8) years.

Table 1 shows sociodemographic variables and disease activity of the two groups of patients at the time of the study visit. No differences between the two groups were found.

Table 2 shows the associations of the 10 polymorphisms with MTX intolerance. The homozygous GG genotype of SNP rs11545078 (GGH-T401C) was significantly less prevalent among intolerant patients than among tolerant patients (77.6% vs. 87.5%, respectively, *p* < 0.05). On the other hand, the homozygous CC genotype of SNP rs717620 (ABCC2-C24T gene) was more frequent among the intolerant patients than among the tolerant patients (51.4% vs. 37.5%, respectively, *p* < 0.05), while the heterozygous TC genotype of the rs717620 polymorphism was less prevalent among the intolerant group (35.5% vs. 50.0%, respectively, *p* < 0.05).

In Table 3, alleles of each polymorphism in these genes were classified into two groups according to the presence of one allele, and the homozygosity of the other base was used as a reference to evaluate the association between MTX intolerance and the allele combination for each polymorphism. These results showed a significant association between MTX intolerance and the GGH-T401C AA or AG genotypes (OR 2.13, 95% CI 1.06–4.29, *p* = 0.035) compared with the GG genotype. On the other hand, an inverse association was observed between the ABCC2-C24T TT or TC genotypes and intolerance to MTX (OR 0.59, 95% CI 0.35–1.00, *p* = 0.05) compared with the CC genotype.

The multivariate logistic regression analysis confirmed the results from the univariate analysis, showing that the GGH-T401C AA or AG and ABCC2-C24T TT or TC genotypes were independently associated with MTX intolerance (OR 2.14, 95% CI 1.05–4.39, *p* = 0.037 and OR 0.54, 95% CI 0.32–0.93, *p* = 0.026, respectively) (Table 4).

## 4. Discussion

MTX represents the first-line treatment for patients with RA, either in monotherapy or in combination with other drugs [14]. MTX is efficacious against both inflammatory symptoms and radiographic destruction [15]; however, intolerance and adverse effects are common and may lead to MTX discontinuation [16]. Therefore, reliable biomarkers to predict MTX intolerance or toxicity in patients with RA should be identified. Our results showed a high prevalence (47%) of intolerant patients compared with values reported in the literature, which may be explained by the use of different definitions of “intolerance”. We considered “intolerant” as a patient who suffered from any type of side effect, while previous studies were focused on a specific type of intolerance or adverse event [11,16].

In our study, we identified one polymorphism associated with MTX intolerance (SNP rs11545078 AA or AG in the gene GGH-T401C) and one polymorphism associated with MTX tolerance (SNP rs717620 TT or TC in the gene ABCC2-C24T). GGH (gamma-glutamyl hydrolase), which is encoded by the GGH gene, is an enzyme that participates in MTX polyglutamate formation, facilitating the elimination of MTX from the cell. Thus, our results suggest that the genetic variant in GGH-T401C (specifically AA or AG) may be associated with MTX intolerance or toxicity in patients with RA. Similarly, previous studies conducted in other RA cohorts indicated that patients with the TT 401C > T genotype showed increased GGH activity compared with patients with the CC or CT genotype, suggesting an association between the TT genotype and an inadequate response to MTX. In addition, a study published by García-Bournissen et al. [17] reported an association between these GGH-T401C alterations and lower enzyme activity, leading to higher hematological MTX toxicity in patients with childhood acute lymphoblastic leukemia [18]. Both studies support our results, suggesting that the presence of this genetic variant (i.e., SNP rs11545078 AA or AG in the GGH-T401C gene) should be investigated in depth as a potential biomarker predicting MTX intolerance.

According to several studies, the MTHFR variants A1298C and C677T are two of the most important polymorphisms associated with MTX toxicity [16,19,20,21]. The MTHFR A1298C polymorphism has been reported to lead to a 60% reduction in enzymatic activity in patients homozygous for 1298C, causing MTX intolerance [22]. In addition, genetic variants causing reduced enzyme activity might contribute to the risk of early increases in alanine aminotransferase (ALT) levels. However, our study did not obtain significant differences related to this polymorphism, which may be explained by the smaller sample size in our study than in these previous studies.

Interestingly, we found that the SNP rs717620 TT or TC genotype from the gene ABCC2-C24T was associated with MTX tolerance. This gene encodes ATP-binding cassette subfamily C member 2, a multispecific organic anion efflux transporter that affects biliary excretion of endogenous and xenobiotic compounds, such as MTX [23]. Based on our results, patients with the T allele exhibited lower toxicity and less intolerance than patients with the CC genotype. These results confirm previous studies showing that patients carrying the T allele exhibit increased MTX clearance, suggesting that ABCC2 is involved in MTX elimination. Hence, the T allele in ABCC2-C24T should be studied in-depth as a potential predictive biomarker for low MTX toxicity.

RFC1-G80A is one of the most analyzed polymorphisms in studies of MTX in patients with RA, but inconsistent results have been published regarding its role in the prediction of the therapeutic response and toxicity. We did not find an association between this polymorphism and MTX intolerance in our patients, suggesting that its effect on MTX outcomes is mild and may be influenced by a combination with some other genetic factors, as described in previous studies [24].

This study has some limitations and some strengths. One limitation is the retrospective nature of the study, as we retrospectively collected information on MTX discontinuation. However, the genetic burden and polymorphisms do not change over time, ensuring the reliability of the genotyping of these patients. The sample size represents another limitation, since our estimation revealed the need for 251 controls (or tolerant patients) and we recruited 120 controls. The sample size may explain why we did not obtain significant differences in some polymorphisms that have been described as associated with MTX intolerance in the literature, such as MTHFR A1298C. One strength of this study is the exclusion of patients who interrupted MTX treatment for reasons other than toxicity or intolerance, such as clinical improvement, inefficacy, or voluntary interruption. In addition, all patients included resided in southern Spain, reducing genetic variability.

## 5. Conclusions

In conclusion, our results show that MTX intolerance is significantly associated with the presence of the AA or AG genotype of the GGH-T401C polymorphism compared with the presence of the GG genotype in a cohort of patients with RA. In addition, we confirmed greater MTX tolerance in carriers of the TT or TC genotype of the ABCC2-C24T polymorphism compared with CC carriers. This study provides new data on the association between polymorphisms and MTX intolerance, which might contribute to the development of new biomarkers and personalized medicine for patients with RA. Future studies conducted in other populations and with greater sample sizes should be conducted to confirm these relationships.

## Figures and Tables

**Table 1:** Sociodemographic characteristics and disease activity at the study visit between tolerant and intolerant patients.

Variable	Intolerant (bDMARDs Monotherapy) Mean (SD) *n* = 107	Tolerant (MTX and bDMARDs Combo Therapy) Mean (SD) *n* = 120	*p*-Value *
Sex (female), *n* (%)	89 (83.2%)	89 (74.2%)	0.100
Age (years)	61.3 (13.2)	58.8 (10.9)	0.133
Age at diagnosis (years)	45.1 (14.0)	43.5 (10.4)	0.344
Moths between diagnosis and MTX initiation	27.6 (65.2)	23.6 (48.9)	0.612
NTD	1.8 (2.8)	1.9 (3.0)	0.888
NSJ	0.7 (2.0)	0.7 (1.5)	0.753
CRP (mg/dL)	1.4 (3.3)	2.8 (7.7)	0.065
ESR (mm/h)	18.6 (16.6)	20.1 (18.6)	0.511
Patient’s VAS (0–10)	3.4 (2.4)	3.7 (2.5)	0.365
Physician’s VAS (0–10)	2.9 (2.1)	2.9 (2.2)	0.967
DAS28 ESR	2.8 (1.1)	2.9 (1.2)	0.589
DAS28 CRP	2.7 (1.1)	2.8 (1.2)	0.355
SDAI	10.2 (8.2)	11.8 (11.8)	0.239
CDAI	8.8 (7.4)	9.0 (7.4)	0.785

* *p*-value for chi-square test or Student *t*-test. CDAI: Clinical Disease Activity Index; CRP: c-reactive protein; DAS28: Disease Activity Score 28; ESR: erythrocyte sedimentation rate; MTX: methotrexate; NTD: number of tender joins; NSJ: number of swollen joints; SDAI: Simple Disease Activity Index; VAS: visual analogue scale.

**Table 2:** Association between methotrexate intolerance and polymorphisms.

Gen (SNP)		Intolerant (bDMARDs Monotherapy) *n* = 107 *n* [% (95% CI)]	Tolerant (MTX and bDMARDs Combo Therapy) *n* = 120 *n* [% (95% CI)]	*p*-Value *
Active Transport of MTX				
RFC-1-G80A (SNP rs1051266)	Homozygotic AA	33 [30.8 (22.1–39.6)]	26 [21.7 (14.3–29.1)]	0.213
	Homozygotic GG	24 [22.4 (14.5–30.3)]	36 [30.0 (21.8–38.2)]	
	Heterozygotic AG	50 [46.7 (37.3–56.2)]	58 [48.3 (39.4–57.2)]	
MTX Polyglutamate Formation				
GGH-T401C (SNP rs11545078)	Homozygotic AA	2 [1.9 (−0.7–4.5)]	1 [0.8 (−0.8–2.4)]	0.138
	Homozygotic GG	83 [77.6 (69.7–85.5)]	105 [87.5 (81.6–93.4)]	0.048
	Heterozygotic AG	22 [20.6 (12.9–28.3)]	14 [11.7 (5.9–17.5)]	0.138
Folate Cycle and Purine Synthesis				
MTHFR-C677T (SNP rs1801133)	Homozygotic AA	15 [14.0 (7.4–20.6)]	22 [18.3 (11.4–25.2)]	0.207
	Homozygotic GG	42 [39.3 (30.1–48.6)]	34 [28.3 (20.2–36.4)]	
	Heterozygotic AG	50 [46.7 (37.3–56.2)]	64 [53.3 (44.4–62.2)]	
MTHFR-A1298C (SNP rs1801131)	Homozygotic TT	50 [45.8 (40.5–49.6)]	66 [55.0 (46.1–63.9)]	0.428
	Homozygotic GG	8 [7.5 (5.1–12.5)]	9 [7.5 (2.8–12.2)]	
	Heterozygotic TG	49 [45.8 (36.4–55.2)]	45 [37.5 (28.8–45.3)]	
DHFR (SNP 1105525)	Homozygotic AA	0 [0 (0–0)]	4 [3.3 (0.1–6.5)]	0.160
	Homozygotic GG	82 [76.6 (68.6–84.6)]	90 [75 (67.3–82.8)]	
	Heterozygotic AG	25 [23.4 (15.4–31.4)]	26 [21.7 (14.3–29.1)]	
SHMT1-c1303C > T (SNP rs1979277)	Homozygotic AA	14 [13.1 (6.7–19.5)]	12 [10.0 (4.6–15.4)]	0.766
	Homozygotic GG	47 [43.9 (34.5–53.3)]	55 [45.8 (38.0–54.7)]	
	Heterozygotic AG	46 [43.0 (33.6–52.4)]	53 [44.2 (35.3–53.1)]	
ITPase-C94A (SNP rs34743033)	Homozygotic AA	2 [1.9 (−0.7–4.5)]	0 [0]	0.294
	Homozygotic GG	93 [86.9 (80.5–93.3)]	104 [86.7 (80.6–92.8)]	
	Heterozygotic AG	12 [11.2 (5.2–17.2)]	16 [13.3 (7.2–19.4)]	
MTX Extraction Pump				
ABCC2-C24T (SNP rs717620)	Homozygotic TT	14 [13.1 (6.7–19.5)]	15 [12.5 (6.6–18.4)]	0.073
	Homozygotic CC	55 [51.4 (41.9–60.9)]	45 [37.5 (28.8–46.2)]	0.035
	Heterozygotic TC	38 [35.5 (26.4–44.6)]	60 [50.0 (51.2–68.7)]	0.035
ABCB1-c3434C > T (SNP rs1045642)	Homozygotic AA	26 [24.3 (16.2–32.4)]	25 [20.8 (13.5–28.1)]	0.801
	Homozygotic GG	31 [29.0 (20.4–37.6)]	38 [31.7 (23.4–40.0)]	
	Heterozygotic AG	50 [46.7 (37.3–56.2)]	57 [47.5 (38.6–56.4)]	
SLCO1B1 (SNP rs11045879)	Homozygotic TT	75 [70.1 (61.4–78.8)]	80 [66.7 (58.3–75.1)]	0.213
	Homozygotic CC	2 [1.9 (0.7–4.5)]	8 [6.7 (2.2–11.2)]	
	Heterozygotic TC	30 [28.0 (19.5–36.5)]	32 [26.7 (18.8–34.6)]	

* *p*-value for chi-square test bDMARDs: biological disease-modifying antirheumatic drugs; CI: Confidence interval; MTX: Methotrexate; OR: Odds Ratio.

**Table 3:** Univariate logistic regression to evaluate the association between MTX intolerance and genetic variants.

Gen (SNP)	Intolerant (bDMARDs Monotherapy) *n* = 107; *n* (%)	Tolerant (MTX and bDMARDs Combo Therapy) *n* = 120; *n* (%)	*p*-Value *	OR (IC 95%)	*p*-Value **
Active Transport of MTX					
RFC-1-G80A GG or AG (vs. AA)	74 (69.2)	94 (78.3)	0.116	0.62 (0.34–1.13)	0.119
MTX Polyglutamate Formation					
GGH-T401C AA or AG (vs. GG)	24 (22.4)	15 (12.5)	0.048	2.13 (1.06–4.29)	0.035
Folate Cycle and Purine Synthesis					
MTHFR-C677T AA or AG (vs. GG)	65 (60.7)	86 (71.7)	0.082	0.61 (0.35–1.07)	0.084
MTHFR–A1298C GG or TG (vs. TT)	99 (92.5)	111 (92.5)	0.995	1.39 (0.82–2.33)	0.219
DHFR AA or AG (vs. GG)	25 (23.4)	30 (25)	0.774	0.87 (0.48–1.60)	0.664
SHMT1-c1303C > T GG or AG (vs. AA)	93 (86.9)	108 (90)	0.466	0.74 (0.33–1.68)	0.470
ITPase-C94A AA or AG (vs. GG)	14 (13.1)	16 (13.3)	0.956	1.06 (0.50–2.26)	0.883
MTX Extraction Pump					
ABCC2-C24T TT or TC (vs. CC)	52 (48.6)	75 (62.5)	0.035	0.59 (0.35–1.00)	0.050
ABCB1-c3434C > T GG or AG (vs. AA)	81 (75.7)	95 (79.2)	0.532	0.78 (0.42–1.45)	0.435
SLC01B1 TT or TC (vs. CC)	105 (98.1)	112 (93.3)	0.107	3.75 (0.78–18.1)	0.099

* chi-square test; ** Univariate logistc regression. bDMARDs: biological disease-modifying antirheumatic drugs; CI: Confidence interval; MTX: Methotrexate; OR: Odds Ratio.

**Table 4:** Multivariate logistic regression to evaluate the association between MTX intolerance and genetic variants (final model).

Gen (SNP)	OR (IC 95%) Intolerant vs. Tolerant	*p*-Value **
GGH-T401C AA or AG (vs. GG)	2.14 (1.05–4.39)	0.037
ABCC2-C24T TT or TC (vs. CC)	0.54 (0.32–0.93)	0.026

** Hosmer–Lemeshow, Chi-square: 1.254, *p* = 0.534.

## Data Availability

Data are available upon a reasonable request.
